# Prevalence and Phenotypic Antimicrobial Resistance of *Listeria monocytogenes* from Animal-Derived Foods: A Scoping Review

**DOI:** 10.3390/foods15183274

**Published:** 2026-09-16

**Authors:** Gina Songor, Miguel Anchundia

**Affiliations:** 1 Master’s Program in Applied Microbiology, Universidad Politécnica Estatal del Carchi, Tulcán 040102, Ecuador; miguel.anchundia@upec.edu.ec; 2 Food Engineering Program, Universidad Politécnica Estatal del Carchi, Tulcán 040102, Ecuador

**Keywords:** *Listeria monocytogenes*, antimicrobial resistance, prevalence, meat products, dairy products, ready-to-eat animal-derived foods, multidrug resistance, food safety

## Abstract

*Listeria monocytogenes* is one of the leading foodborne pathogens and an increasing public health concern because of the emergence of antimicrobial-resistant strains. This scoping review aimed to synthesize the available evidence on the prevalence and phenotypic antimicrobial resistance profiles of *L. monocytogenes* in animal-derived foods. The review was conducted following the Joanna Briggs Institute methodology and the PRISMA-ScR guidelines through systematic searches of PubMed/MEDLINE, Scopus, Web of Science, and Embase. Of the 711 records identified, 11 studies met the eligibility criteria. The included studies reported the occurrence of *L. monocytogenes* in meat and meat products, milk and dairy products, and ready-to-eat foods. The reported prevalence ranged from 0.1% to 60.0% across food matrices; however, the highest estimate (60.0%) was derived from a single ready-to-eat food study with a small sample size (9 positive samples among 15 analyzed) and should therefore be interpreted cautiously. Phenotypic resistance was most frequently reported to specific β-lactams, particularly penicillin, oxacillin, cloxacillin, cefotaxime, ceftriaxone, and piperacillin, as well as to tetracyclines. In contrast, several studies reported preserved susceptibility to ampicillin and amoxicillin, indicating substantial variability in resistance within the β-lactam class across the included studies. This scoping review provides an updated synthesis of the available evidence on the prevalence and phenotypic antimicrobial resistance profiles of *L. monocytogenes* in animal-derived foods and summarizes the phenotypic antimicrobial resistance patterns reported across the included food matrices.

## 1. Introduction

Listeriosis is a severe foodborne disease caused by the Gram-positive bacterium *L. monocytogenes*. According to the most recent European Union One Health Zoonoses Report, 3041 confirmed invasive human cases of listeriosis were reported in the European Union in 2024, corresponding to a notification rate of 0.69 cases per 100,000 population. Despite its relatively low incidence compared with other foodborne zoonoses, listeriosis was associated with the highest proportions of hospitalizations and deaths, reaching 97.3% and 15.6%, respectively, among non-outbreak-related infections. Moreover, a significant increasing trend in reported listeriosis cases has been observed over the previous five years [1].

These epidemiological data highlight the substantial public health impact of *L. monocytogenes*, particularly among vulnerable populations, including pregnant women, neonates, older adults, and immunocompromised individuals [2,3]. Because of its clinical and epidemiological impact, *L. monocytogenes* is regarded as a high-priority pathogen for surveillance and food safety.

The importance of this microorganism lies in its ability to survive as an environmental saprophyte and subsequently transition into an intracellular pathogen following the consumption of contaminated food, overcoming host physiological barriers through complex cellular interactions [4]. This environmental-to-host transition contributes to its establishment as a persistent foodborne pathogen. In food-processing environments, persistence can be defined as the ability of a microorganism to become established in specific niches or harborage sites for prolonged periods, despite the frequent application of cleaning and disinfection procedures [5]. In *L. monocytogenes*, persistence is commonly evidenced by the repeated detection of genetically indistinguishable or closely related strains in the same processing environment over time [6]. Its persistence throughout food production systems is further supported by its remarkable ability to adapt to environmental stress, allowing survival at refrigeration temperatures and tolerance to a variety of disinfectants commonly used in the food industry [7].

*Listeria monocytogenes* can establish biofilms on hard-to-clean niches, where the extracellular polymeric matrix protects bacterial cells from cleaning and disinfection procedures and facilitates cross-contamination during food processing [3,7]. Consequently, even when sanitation procedures are properly implemented, the persistence of this pathogen may increase the likelihood of recurrent food contamination, thereby increasing the potential for consumer exposure [8].

Beyond the clinical implications of listeriosis, increasing attention has been directed toward the emergence of antimicrobial-resistant isolates. The first-line treatment for listeriosis has traditionally been ampicillin or penicillin G, often combined with gentamicin. However, the emergence of antimicrobial-resistant isolates has become a growing public health concern because of its potential impact on therapeutic efficacy. Although most *L. monocytogenes* isolates remain susceptible to first-line antimicrobial agents, the intensive and inappropriate use of antibiotics in the clinical, veterinary, and agricultural sectors creates selective pressure that favors the emergence and dissemination of resistant strains throughout the food chain. This process contributes to the dissemination of antimicrobial resistance determinants among bacterial populations and represents a growing challenge. The development of antimicrobial resistance in *L. monocytogenes* is a dynamic process driven by several molecular mechanisms, including intrinsic and acquired resistance. The latter may occur through horizontal gene transfer mediated by plasmids and transposons, facilitating the acquisition of resistance determinants such as *tet* genes associated with tetracycline resistance [9,10].

Acquired resistance in *L. monocytogenes* may occur through horizontal gene transfer mediated by mobile genetic elements, including plasmids and transposons. Several plasmids have been described in *L. monocytogenes*, including pNH1, p900703_1, pLM466-3, pLMR479a, pLM1686, pLM80, pLM6179, pLM5578, pLM58, and pMF4545. Among these, pNH1, p900703_1, and the recently described hybrid plasmid pLM466-3 are particularly relevant to antimicrobial resistance because they harbor multiple resistance determinants. Plasmid pNH1 carries genes associated with resistance to tetracyclines, macrolides, lincosamides, aminoglycosides, trimethoprim, and chloramphenicol, whereas p900703_1 harbors resistance determinants including *dfrD*, *lnuG*, and *mphB*. In addition, pLM466-3, reported in foodborne *L. monocytogenes* in 2026, represents a hybrid multidrug-resistance plasmid related to pNH1 and pLMR479a. Other plasmids, including pLM80, pLM6179, pLM5578, pLM58, pMF4545, and pLM1686, have been associated mainly with environmental adaptation and stress tolerance, which may contribute indirectly to the survival and persistence of *L. monocytogenes* in food-processing environments [11,12].

Several studies have documented resistance to multiple antimicrobial classes among *L. monocytogenes* isolates recovered from foods. Resistance to tetracyclines has been reported in several studies [13,14,15], whereas fluoroquinolone resistance has also been documented [14,16]. Similarly, lincosamide resistance has been reported [13,15,17]. In addition, multidrug-resistant (MDR) profiles have been identified in isolates recovered from different food matrices [13,14,18], demonstrating the occurrence of multidrug resistance among foodborne *L. monocytogenes* isolates. Collectively, these findings highlight the importance of strengthening microbiological and antimicrobial resistance surveillance in foods.

Despite the growing number of studies investigating the occurrence of *L. monocytogenes* in animal-derived foods and their antimicrobial resistance profiles, the available scientific evidence remains scattered across different geographic regions, food matrices, and methodological approaches. Moreover, existing studies vary in the types of foods evaluated, bacterial isolation and identification methods, antimicrobial susceptibility testing procedures, and antimicrobial agents analyzed, making direct comparisons among studies difficult and hampering the identification of consistent resistance patterns [2]. This methodological heterogeneity, together with differences in food matrices and geographical settings, complicates the interpretation and comparison of the prevalence and antimicrobial resistance profiles of *L. monocytogenes* across studies [19].

Consequently, there is a need to synthesize and integrate the available evidence to provide a comprehensive overview of the occurrence of *L. monocytogenes* and its antimicrobial resistance patterns across different food matrices, while providing an evidence base to support microbiological monitoring, food safety risk assessment, and control strategies throughout the food production systems.

Within this context, a scoping review was conducted following the PRISMA-ScR guidelines to synthesize the available evidence on the prevalence and phenotypic antimicrobial resistance profiles of *L. monocytogenes* in animal-derived foods. The review aimed to characterize antimicrobial resistance patterns across different food matrices and provide an evidence base to support coordinated microbiological monitoring, food safety risk assessment, and evidence-based decision-making within a One Health framework.

## 2. Materials and Methods

### 2.1. Study Design and Protocol Registration

This scoping review was developed a priori and conducted in accordance with the Preferred Reporting Items for Systematic Reviews and Meta-Analyses Extension for Scoping Reviews (PRISMA-ScR) guidelines [20], to enhance the transparency, reproducibility, and methodological rigor of the review process. The study protocol was prospectively registered in the Open Science Framework (OSF) with the registration identifier osf.io/7xcwf.

### 2.2. Research Question

The research question was formulated using the Population–Concept–Context (PCC) framework recommended by the Joanna Briggs Institute for the development of scoping reviews. This framework was used to define the scope of the review and establish the eligibility criteria for study selection, as follows:

- P (Population): *L. monocytogenes* isolates.
- C (Concept): Phenotypic antimicrobial resistance.
- C (Context): Meat, meat products, raw milk, ready-to-eat animal-derived foods and dairy products.

What scientific evidence is available regarding the prevalence and phenotypic antimicrobial resistance profiles of *L. monocytogenes* isolated from meat, meat products, raw milk, ready-to-eat animal-derived foods and dairy products?

### 2.3. Systematic Literature Search

A systematic literature search was conducted in the electronic databases PubMed/MEDLINE, Scopus, Web of Science, and Embase, covering all studies published from database inception through 27 December 2025. In addition, a manual search of the reference lists of the included articles and other relevant publications was performed to identify potentially eligible studies not identified through the electronic search strategy.

The general search strategy was developed around the main concepts of *L. monocytogenes*/listeriosis, antimicrobial resistance, foodborne transmission and foodborne diseases, public health impact, and antimicrobial resistance mechanisms. Based on these concepts, the general search framework was structured as follows:

(*Listeria monocytogenes* OR listeriosis) AND (“antimicrobial resistance” OR “antibiotic resistance” OR “drug resistance”) AND (foodborne OR “foodborne disease” OR “foodborne illness” OR “food poisoning”).

The search strategy was adapted to the terminology and syntax of each database, using Medical Subject Headings (MeSH) for PubMed/MEDLINE, Emtree terms for Embase, and free-text keywords across all databases. Health Sciences Descriptors (DeCS) were used to verify terminological equivalence during the development of the search strategy but not as controlled vocabulary in the international databases.

Searches were conducted using the search interface provided by each database. Depending on the search functionality of each database, each conceptual block was entered into separate search fields (e.g., Title, Abstract, Keywords, Topic, MeSH, or Emtree) and combined using the Boolean operators AND and OR. The complete search strategies presented in Table 1 correspond to the Boolean expressions derived from the search concepts entered through these interfaces. No restrictions were applied regarding language or publication date.

### 2.4. Study Selection

All records identified through the database searches were initially imported into Mendeley Reference Manager (version 2.148.0) for reference management and subsequently exported to Rayyan (https://www.rayyan.ai/, accessed on 29 December 2025), where duplicate records were identified and removed, and the study screening process was conducted.

Study selection was performed in two sequential stages. In the first stage, the two authors (G.S. and M.A.) independently assessed the publication types, as well as the titles and abstracts of the retrieved records, according to the predefined eligibility criteria to identify potentially relevant studies, thereby minimizing selection bias. Articles that did not meet the inclusion criteria were excluded at this stage.

In the second stage, the full texts of the potentially eligible studies were retrieved, and both authors independently assessed their eligibility according to the predefined inclusion and exclusion criteria (Table 2). The reasons for excluding studies assessed at the full-text stage were systematically documented according to the predefined eligibility criteria. Any disagreements between the reviewers were resolved through consensus. Finally, only studies reporting data on the antimicrobial susceptibility or resistance of *L. monocytogenes* isolated from meat, meat products, raw milk, dairy products, or ready-to-eat animal-derived foods were included in the qualitative synthesis.

The entire study selection process, including records identified, duplicate records removed, records screened, full-text reports assessed for eligibility, studies excluded, and studies included, were documented using the PRISMA 2020 flow diagram in accordance with the PRISMA-ScR reporting recommendations to enhance the transparency and reproducibility of the review process.

The eligibility criteria applied during study selection are summarized in Table 2. Eligible sources were original observational studies reporting primary data on the isolation and identification of *L. monocytogenes* from meat, meat products, raw milk, dairy products, or ready-to-eat animal-derived foods and providing phenotypic antimicrobial susceptibility or resistance results. Studies were required to provide sufficient methodological information regarding bacterial identification and antimicrobial susceptibility testing. Narrative reviews, systematic reviews, meta-analyses, editorials, letters to the editor, conference abstracts, case reports, book chapters, experimental studies using reference strains or artificially inoculated samples, studies not reporting antimicrobial susceptibility or resistance data, and studies focusing exclusively on environmental samples unrelated to eligible food matrices were excluded. No restrictions were applied regarding language or publication date. Publications in languages other than English were eligible for screening and, when necessary, were translated into American English for eligibility assessment and data extraction. All studies ultimately included in this scoping review were available in English.

### 2.5. Data Extraction

Data extraction was performed using a standardized, predefined data extraction form to systematically record the methodological characteristics and relevant outcome data of the included studies, as well as variables related to the prevalence and antimicrobial resistance of *L. monocytogenes*. The following information was extracted from each study: first author; year of publication; study design; country where the study was conducted; isolation source; food sample type; sample size; methods used for bacterial isolation and identification; antimicrobial susceptibility testing method; antimicrobial agents evaluated; reported prevalence, when available; and antimicrobial resistance profiles (File S1: Optimized_Matrix_Listeria).

Data extraction was performed independently by both investigators (G.S. and M.A.) using a structured, predefined data extraction matrix developed according to the objectives of the review and the variables described above. Any discrepancies were resolved through joint reassessment of the full text of the corresponding study, with the extracted data verified against the predefined fields of the data extraction matrix. Any remaining differences were discussed until consensus was reached.

The extracted data were subsequently organized into structured tables to facilitate comparisons across the included studies and support the narrative synthesis of the included evidence. This approach facilitated a comprehensive description of the methodological characteristics of the studies, the prevalence of *L. monocytogenes*, and the antimicrobial resistance patterns identified across the different food matrices evaluated.

### 2.6. Data Synthesis

A qualitative descriptive synthesis of the included studies was conducted, with the extracted data organized into tables to summarize study characteristics and facilitate comparisons across the included evidence. The synthesis was carried out in three stages: (i) description of the included studies, (ii) analysis of the prevalence of *L. monocytogenes* in foods, and (iii) assessment of the antimicrobial resistance of the isolates. Findings were compared across food matrices, including meat, meat products, raw milk, dairy products, and ready-to-eat animal-derived foods and, whenever possible, according to study characteristics and the classes of antimicrobial agents evaluated.

Because of the methodological heterogeneity of the included studies with respect to study design, food matrices, microbiological techniques, antimicrobial susceptibility testing methods, and the reporting of results, the evidence was synthesized using a narrative approach, identifying similarities, differences, and reported antimicrobial resistance patterns across the included studies. The findings were presented in comparative tables to facilitate their interpretation.

For the antimicrobial resistance heatmap, when multiple antimicrobial agents belonging to the same class were evaluated within a study, the highest resistance percentage reported for an individual antimicrobial within that class was used as the class-level value. Thus, the heatmap represents the maximum reported phenotypic resistance within each antimicrobial class rather than the average resistance across agents.

### 2.7. Critical Appraisal of the Sources of Evidence

In accordance with the objectives of scoping reviews and the recommendations of the PRISMA-ScR reporting guidelines and the Joanna Briggs Institute (JBI), no formal methodological quality assessment or risk-of-bias assessment of the included studies was performed, as the purpose of this review was to map and synthesize the available evidence on the prevalence and antimicrobial resistance profiles of *L. monocytogenes* isolated from animal-derived foods rather than to evaluate the quality of the evidence.

## 3. Results

### 3.1. General Description of the Included Studies

**Figure 1:**
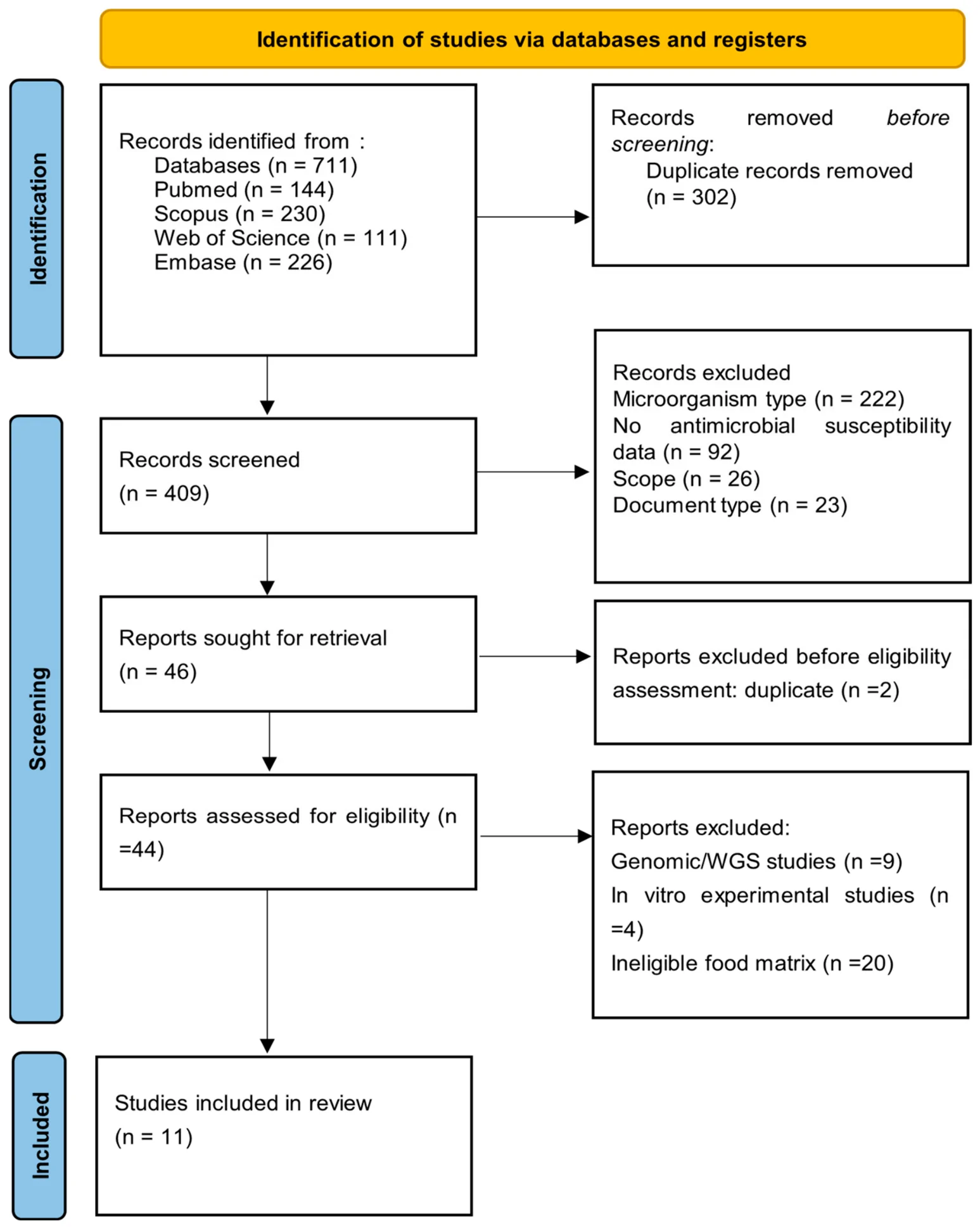
PRISMA 2020 flow diagram of the study selection process.

A total of 711 records were identified through PubMed (*n* = 144), Scopus (*n* = 230), Web of Science (*n* = 111), and Embase (*n* = 226). After removal of 302 duplicate records, 409 unique records underwent title and abstract screening, of which 363 were excluded. Consequently, 46 reports were sought for full-text retrieval. Two reports were subsequently excluded because they represented duplicate publications, leaving 44 reports for full-text eligibility assessment. Of these, 33 reports were excluded because they did not meet the predefined eligibility criteria: genomic/WGS studies (*n* = 9), in vitro experimental studies (*n* = 4), and studies involving ineligible food matrices (*n* = 20). Ultimately 11 studies were included in the qualitative synthesis (Figure 1).

The included studies were published between 2014 and 2025, encompassing more than a decade of research on the occurrence and antimicrobial resistance of *L. monocytogenes* in animal-derived foods. The studies were conducted in seven countries, including Nigeria [13], Romania [14], India [16,17,21], Iran [15,22], Egypt [23], Ethiopia [24,25] and Portugal [18], reflecting the global interest in investigating *L. monocytogenes* in animal-derived foods (Table 3).

The food matrices investigated primarily included raw beef [13,15,17,21,24], raw goat meat and chicken meat [13], raw poultry meat [14], meat products [13,15,18], raw bovine milk [21,25], milk and dairy products [15,22], as well as ready-to-eat foods [16], demonstrating the broad range of food matrices evaluated across the included studies (Table 3).

The number of samples varied considerably across the included studies, ranging from 15 to 765 samples. The smallest sample size was reported by Gautam et al. [16], who analyzed 15 ready-to-eat food samples, whereas the largest was reported by Gowda et al. [17], with 765 samples. The remaining studies included between 75 and 545 samples [13,14,15,16,17,18,21,22,23,24,25], reflecting variations in study scope and sampling strategies.

### 3.2. Prevalence of L. monocytogenes in Food Matrices

The prevalence of *L. monocytogenes* reported in the included studies varied widely, ranging from 0.1% to 60% across different food matrices and sample sizes (Table 4). The highest prevalence (60%) was reported in a single study of ready-to-eat foods based on a small sample size (9 positive samples among 15 analyzed); therefore, this estimate should be interpreted cautiously and should not be considered representative of ready-to-eat foods in general.

Among meat and meat product matrices, the prevalence of *L. monocytogenes* ranged from 0.1% to 16%. The lowest prevalence was observed in raw beef (0.1–4.4%) [17,21,24], whereas higher values were reported in raw chicken meat (13.2%) [14] and in raw beef, raw pork, meatballs, hamburgers, fresh sausages, breaded meat skewers, and traditional Portuguese sausages (Alheira and Moura) (16%) [18]. Similarly, studies evaluating raw beef, goat meat, chicken meat, suya, Kilishi, Balangu, and Tsire [13], as well as sausages, pasteurized milk, cheese, raw chicken meat, and beef, goat, and sheep meat cubes [15], reported intermediate prevalence values of 4% and 3%, respectively, indicating that *L. monocytogenes* was detected across a wide range of animal-derived food matrices.

Among dairy matrices, the reported prevalence of *L. monocytogenes* ranged from 1.1% to 7%, with the lowest reported prevalence observed in raw bovine milk (1.1%) [21] and the highest in commercially marketed raw bovine milk (7%) [23]. In addition, one study evaluating raw milk, cheese, butter, curd, and ice cream reported an intermediate prevalence of 4% [22], indicating the prevalence of *L. monocytogenes* across a broad range of dairy products.

Among ready-to-eat food matrices, only one study evaluated this category, including a variety of meat- and vegetable-based ready-to-eat foods, and reported the highest prevalence observed among the studies included (60%) [16]. This was the highest prevalence reported across all food matrices evaluated in this review.

### 3.3. Antimicrobial Resistance Profiles of L. monocytogenes

Antimicrobial resistance was reported across all 11 included studies, although the resistance patterns varied according to the food matrix and the antimicrobial agents evaluated (Table 5). Resistance to β-lactams was the most frequently reported finding, being documented in 10 of the 11 studies [13,15,16,18,21,23,24,25]. Resistance to tetracyclines was reported in 8 studies [13,14,18,22,23,24,25], whereas resistance to sulfonamides, quinolones/fluoroquinolones, macrolides, aminoglycosides, and phenicols was reported less consistently across the included evidence. Multidrug-resistant (MDR) isolates were identified in 8 studies [13,14,16,18,21,22,23,24], although the reported frequency varied considerably among food matrices and geographic regions.

Resistance to β-lactams was the most frequently reported antimicrobial resistance pattern and was observed in isolates recovered from beef, goat, and chicken meat, traditional meat products, raw milk, cheese, butter, curd, ice cream, and ready-to-eat foods [13,15,16,21,22,24,25]. The antimicrobial agents most frequently associated with resistance were penicillin, ampicillin, oxacillin, cefotaxime, ceftriaxone, piperacillin, and cloxacillin. Notably, 100% resistance to penicillin G, piperacillin, oxacillin, and ceftriaxone was reported among isolates from raw bovine milk [21]. Likewise, isolates recovered from raw beef exhibited high resistance frequencies to oxacillin and cefotaxime [24], whereas isolates from ready-to-eat foods also showed resistance to cefotaxime and oxacillin [16].

Tetracyclines represented the second-most frequently reported class exhibiting resistance. This pattern was identified in isolates recovered from fresh meat, meat products, raw milk, dairy products, and ready-to-eat foods [13,14,18,22,23,24,25]. Similarly, resistance to sulfonamides and trimethoprim/sulfamethoxazole was observed primarily in meat and meat products [13,14,18], whereas resistance to quinolones and fluoroquinolones, including ciprofloxacin, levofloxacin, and nalidixic acid, was reported in meat, milk, and ready-to-eat foods [14,16,18,24,25]. Resistance to macrolides, aminoglycosides, and phenicols was also reported, although less frequently and with greater variability across the different food matrices [16,17,18,22,23,24].

In contrast, several studies reported high susceptibility to vancomycin, linezolid, gentamicin, amoxicillin, ampicillin, and chloramphenicol among isolates recovered from both meat and dairy products [13,15,17,18,21,22,24,25]. High susceptibility to these antimicrobial agents was reported across several included studies, despite resistance to other β-lactams and tetracyclines in different studies, highlighting substantial inter-study and antimicrobial-specific variability.

Multidrug resistance (MDR) was reported in several studies; however, the definitions of MDR, antimicrobial panels, and interpretative criteria were not uniform across the included investigations. Among isolates recovered from raw chicken meat, 23% were reported as MDR [14], whereas a frequency of 28.6% was reported among isolates from beef, pork, and meat products [18]. Other studies reported MDR frequencies of 100% among isolates from raw bovine milk [21] and 95% among isolates from raw beef [24]. In addition, resistance to multiple antimicrobial agents was reported among isolates from dairy products [22,23]. In contrast, Lotfollahi et al. [15] and Gowda [17] did not report MDR isolates. Therefore, MDR frequencies reported across the included studies should be interpreted individually rather than directly compared.

**Figure 2:**
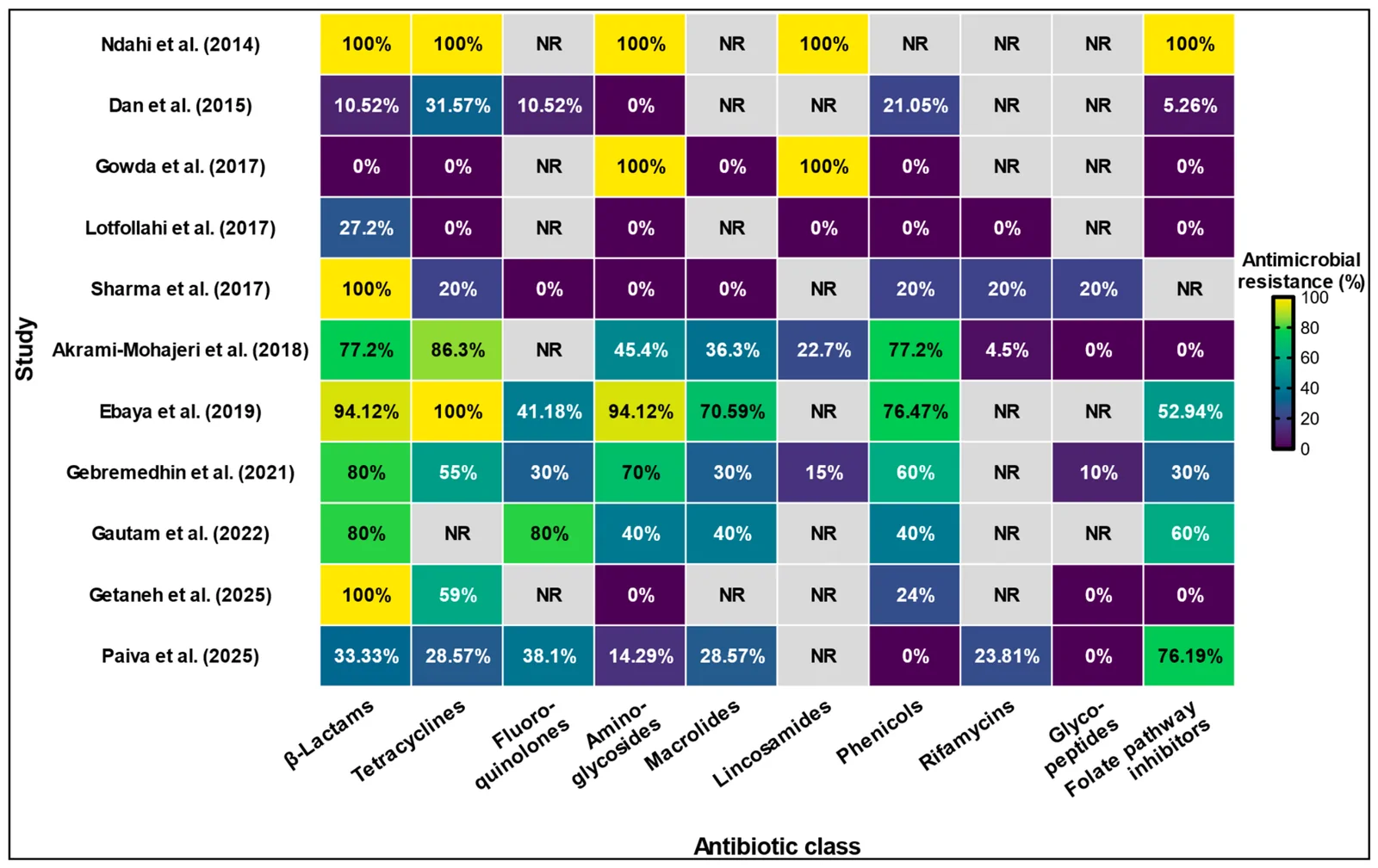
Heatmap of phenotypic antimicrobial resistance profiles of *L. monocytogenes* across the included studies [13,14,15,16,17,18,21,22,23,24,25]. The highest resistance percentage reported for an individual agent was used to represent the corresponding antimicrobial class when multiple antimicrobial agents within the same class were evaluated in a study. NR indicates that resistance to the corresponding antimicrobial class was not reported.

The heatmap of antimicrobial classes (Figure 2) indicates substantial heterogeneity in phenotypic antimicrobial resistance across the included studies. β-Lactams showed the highest resistance percentages in several studies, while tetracyclines also exhibited notable resistance levels. In contrast, resistance to aminoglycosides, fluoroquinolones, and macrolides was more variable across studies. These patterns should be interpreted with the understanding that the values represent the highest resistance percentage reported for an individual antimicrobial agent within each class.

## 4. Discussion

The objective of this scoping review was to synthesize and map the available scientific evidence on the prevalence of *L. monocytogenes* and its phenotypic antimicrobial resistance profiles in animal-derived foods intended for human consumption. The available evidence indicates that *L. monocytogenes* remains a major foodborne pathogen because of its ability to persist throughout different stages of the food chain and to exhibit resistance to clinically important antimicrobial agents. This evidence reinforces its continued relevance, particularly because invasive listeriosis is associated with high hospitalization and mortality rates among pregnant women, neonates, older adults, and immunocompromised individuals [2,3,4].

The evidence gathered also indicates that antimicrobial resistance in *L. monocytogenes* should be interpreted within an integrated One Health framework, as the circulation of resistant strains involves animal production, food processing, the environment, and human health. The food chain may therefore act as both a reservoir and a dissemination route for antimicrobial resistance determinants, highlighting the need for coordinated surveillance and control strategies across the veterinary, food, and environmental sectors [19].

Although antimicrobial-resistant *L. monocytogenes* was identified across different food matrices, these findings should be interpreted cautiously because substantial heterogeneity was observed in sampling strategies, isolation and identification methods, antimicrobial susceptibility testing protocols, and interpretative criteria. Consequently, this review provides an updated synthesis of the available evidence and identifies overall resistance patterns but does not allow reliable estimation of the global burden of antimicrobial resistance in *L. monocytogenes*. These limitations emphasize the need to harmonize study methodologies and susceptibility testing approaches to improve international comparability and strengthen global epidemiological surveillance.

The reported prevalence of *L. monocytogenes* varied markedly among the included studies, ranging from 0.13% to 60.00%. The lowest prevalence (0.13%) was reported by Gowda et al. [17] in samples collected from slaughterhouses and cattle production environments, whereas the highest prevalence (60.00%) was observed by Gautam et al. [16] in ready-to-eat foods. This wide variation probably reflects differences in food matrices, sampling strategies, processing conditions, and laboratory detection methods, all of which influence the survival, persistence, and recovery of *L. monocytogenes* throughout the food chain.

From a microbiological perspective, the ability of *L. monocytogenes* to survive in different food matrices depends on intrinsic factors such as pH, water activity, nutrient availability, and food composition, together with extrinsic factors including storage temperature, processing conditions, and hygienic practices during production and handling. These factors directly influence pathogen survival, growth, and recovery during microbiological analysis and may partly explain the differences in prevalence observed among meat, dairy products, and ready-to-eat foods [26,27,28,29,30].

Dairy products remain one of the principal food matrices associated with *L. monocytogenes* contamination. The recurrent detection of the pathogen in raw milk, pasteurized milk, soft cheeses, and other dairy products [15,21,22,23] indicates that these foods continue to represent an important source of consumer exposure. This persistence has been attributed to the ability of *L. monocytogenes* to survive and grow under refrigeration, form biofilms on food-processing surfaces, and adapt to a wide range of environmental stresses, thereby facilitating long-term establishment within dairy processing environments and increasing the risk of cross-contamination during production [31].

Ready-to-eat foods also represent an important public health concern because they are consumed without a subsequent heat treatment capable of eliminating *L. monocytogenes*. The high prevalence reported by Gautam et al. [16], for ready-to-eat foods (60%; 9/15 samples) should be interpreted cautiously because it was derived from a single study with a small sample size, limiting its generalizability to the broader ready-to-eat food category. Nevertheless, the overall pattern is consistent with previous evidence identifying ready-to-eat foods as one of the principal routes of human exposure to *L. monocytogenes* and a priority target for food safety surveillance programs [30,32,33,34].

Overall, the marked variability in the reported prevalence indicates that contamination by *L. monocytogenes* does not follow a uniform epidemiological pattern but instead reflects differences in production systems, food processing, commercialization practices, and pathogen ecology. Consequently, prevalence should not be interpreted as an isolated indicator of public health risk, since a low isolation frequency does not exclude the presence of strains with enhanced persistence, environmental adaptation, or virulence potential. Instead, prevalence should be interpreted within the broader context of food characteristics, production practices, and pathogen biology.

The phenotypic antimicrobial resistance profiles of *L. monocytogenes* isolates exhibited substantial variability across the included studies. Despite this heterogeneity, resistance was most consistently reported against β-lactams and tetracyclines, whereas susceptibility and resistance patterns for the remaining antimicrobial classes varied considerably among isolates recovered from meat, meat products, milk, dairy products, and ready-to-eat foods [13,14,15,16,18,21,22,35].

The predominance of resistance to β-lactams and tetracyclines is consistent with recent systematic reviews and meta-analyses identifying these antimicrobial classes as the most frequently associated with resistance among foodborne *L. monocytogenes* isolates [36,37,38]. Although resistance frequencies differed among geographical regions and food matrices, the recurrence of this pattern suggests that resistance to these antimicrobial classes is widespread rather than restricted to specific epidemiological settings.

β-Lactams represented the antimicrobial class most frequently associated with resistance among the included studies. The detection of β-lactam-resistant *L. monocytogenes* isolates in raw meat, meat products, milk, and cattle [13,15,21,23,25] is particularly relevant because aminopenicillins remain the first-line treatment for invasive listeriosis [3,27]. Consequently, resistance to these antimicrobial agents should be regarded as an important epidemiological warning signal requiring continuous monitoring, even when resistant isolates are recovered from foods rather than clinical cases.

However, the clinical significance of β-lactam resistance should be interpreted according to the specific antimicrobial agents evaluated. Whereas resistance to penicillin and ampicillin may have direct therapeutic implications, the high resistance rates reported for cefotaxime, ceftriaxone, and other cephalosporins should not be interpreted as evidence of acquired resistance. *L. monocytogenes* is intrinsically less susceptible to cephalosporins because of the low affinity of specific penicillin-binding proteins for these antimicrobial agents. Consequently, grouping all β-lactams into a single category may overestimate the clinical relevance of the observed resistance pattern [39].

Tetracycline resistance was also one of the most consistent findings of this review. The resistance profiles identified among *L. monocytogenes* isolates recovered from meat, meat products, milk, and dairy products [15,18,22,23] indicate that resistance to this antimicrobial class remains widely distributed across different food production systems. This pattern is generally attributed to the historical selective pressure associated with tetracycline use in food-producing animals, although the included studies did not directly evaluate this relationship.

These findings agree with recent systematic reviews identifying tetracyclines among the antimicrobial classes most frequently associated with resistance in foodborne *L. monocytogenes* isolate [37,38,40,41]. Unlike the intrinsically reduced susceptibility observed for certain cephalosporins, tetracycline resistance is primarily mediated by acquired and transferable determinants, particularly the ribosomal protection genes tetM and tetS, together with efflux mechanisms. These genetic determinants facilitate the dissemination and maintenance of tetracycline resistance across different bacterial reservoirs, reinforcing their epidemiological importance [38,42].

Experimental transfer of the *tetM* gene from a food-derived *L. monocytogenes* isolate to *Enterococcus faecalis* in processed cheese further suggests that food matrices may support the horizontal transfer of antimicrobial resistance genes under favorable conditions [42]. Although most studies included in this review evaluated only phenotypic resistance and did not investigate the underlying molecular mechanisms, the available evidence provides a biologically plausible explanation for the recurrent occurrence of tetracycline resistance among foodborne *L. monocytogenes* isolates.

Fluoroquinolones, macrolides, aminoglycosides, sulfonamides, and phenicols exhibited considerably more heterogeneous resistance patterns across the included studies. While some investigations reported high resistance frequencies, others documented low or no resistance to the same antimicrobial classes, highlighting substantial variability among food matrices, geographical regions, production systems, and the distribution of acquired resistance determinants [38,41]. Moreover, recent genomic studies have demonstrated that the presence of resistance genes does not always translate into phenotypic resistance, emphasizing the complexity of resistance regulation and expression in *L. monocytogenes* [43].

Despite the resistance reported for several antimicrobial classes, many studies consistently documented high susceptibility to ampicillin, amoxicillin, vancomycin, linezolid, and gentamicin among *L. monocytogenes* isolates recovered from animal-derived foods [13,15,17,18,21,22,24,25]. These findings indicate that resistance to these clinically important antimicrobial agents remains relatively uncommon among foodborne isolates [37,38,44]. Nevertheless, the coexistence of susceptible and resistant isolates highlights the dynamic nature of antimicrobial susceptibility and underscores the importance of continuous surveillance to detect emerging resistance patterns, monitor temporal changes, and support evidence-based management of listeriosis.

An important factor that may partly explain the variability in antimicrobial resistance frequencies observed across the included studies is the lack of uniformity in antimicrobial susceptibility testing methods and interpretative criteria. The studies included in this review were conducted over different periods and applied different antimicrobial panels, testing procedures, and interpretative standards, including Standards established by the Clinical and Laboratory Standards Institute (CLSI) and the European Committee on Antimicrobial Susceptibility Testing (EUCAST) criteria [45,46].

This is particularly relevant for *L. monocytogenes*, because species-specific clinical breakpoints are not available for all antimicrobial agents evaluated in foodborne isolates. Consequently, some studies have interpreted susceptibility using breakpoints established for other bacterial species or groups when *L. monocytogenes*-specific criteria were unavailable. Such methodological differences may result in the same or similar MIC values or inhibition-zone diameters being classified differently depending on the interpretative criterion applied. Therefore, differences in the reported resistance frequencies across studies should not necessarily be interpreted as true epidemiological differences in antimicrobial resistance, as they may partly reflect differences in testing methodology, breakpoint selection, and the version of the interpretative standard used [38,39,43].

This limitation is particularly important when comparing resistance frequencies across countries, food matrices, and study periods. Future studies should therefore report the antimicrobial susceptibility testing method, antimicrobial concentrations or disk contents, the interpretative standard and version used, and the specific clinical breakpoint or epidemiological cutoff applied. Greater harmonization of these methodological criteria would improve comparability among studies and allow more robust surveillance of phenotypic antimicrobial resistance in foodborne *L. monocytogenes*.

Multidrug resistance (MDR) was one of the most relevant findings identified in this review. Several studies reported *L. monocytogenes* isolates resistant to three or more antimicrobial classes recovered from meat, meat products, milk, and ready-to-eat foods [15,18,21,22,23,24,25]. Although MDR frequencies varied substantially across studies, the recurrent detection of multidrug-resistant isolates in different food matrices indicates that MDR is widely distributed among foodborne *L. monocytogenes* and remains an important challenge for food safety.

The available evidence is consistent with recent systematic reviews reporting the recurrent detection of multidrug-resistant *L. monocytogenes* isolates in foods of animal origin across different geographical regions [37,38,44]. Nevertheless, direct comparisons among studies should be interpreted cautiously because MDR definitions, antimicrobial susceptibility testing panels, and interpretative criteria were not standardized across the investigations included in this review.

The emergence of multidrug-resistant *L. monocytogenes* has been primarily attributed to the accumulation of multiple antimicrobial resistance determinants within the same bacterial isolate. These determinants are frequently carried by plasmids, transposons, and other mobile genetic elements, facilitating the acquisition and dissemination of resistance to different antimicrobial classes [38,42,43].

Horizontal gene transfer may further contribute to the dissemination of multidrug resistance, as resistance genes associated with tetracyclines, macrolides, and other antimicrobial classes can be exchanged between *L. monocytogenes* and other Gram-positive bacteria present in foods or food-processing environments [38,42]. This mechanism reinforces the role of the food-processing environment as a potential reservoir and dissemination route for antimicrobial resistance determinants.

From a food safety perspective, the recurrent detection of multidrug-resistant *L. monocytogenes* isolates highlights the need to strengthen antimicrobial resistance surveillance throughout the food chain. Integrating microbiological monitoring, prudent antimicrobial use, and molecular characterization could facilitate the early detection and tracking of resistant strains, thereby supporting more effective systematic monitoring and evidence-based food safety risk management [43,47].

The available evidence on multidrug resistance should nevertheless be interpreted cautiously because substantial methodological heterogeneity was observed among the included studies. Differences in MDR definitions, antimicrobial panels, and susceptibility interpretative criteria limit direct comparisons across investigations and probably contribute to the reported variability. Consequently, the burden of MDR should always be interpreted within the methodological context of each study and the characteristics of the food matrices evaluated.

Beyond antimicrobial resistance, the long-term persistence of *L. monocytogenes* within food-processing environments represents another major factor contributing to its public health significance. The organism can survive on equipment, food-contact surfaces, drains, and other difficult-to-clean niches, where it withstands refrigeration temperatures, nutrient limitation, cleaning procedures, and other environmental stresses [31,48,49,50]. These adaptive traits facilitate cross-contamination and contribute to the long-term establishment of the pathogen within food-processing facilities.

Biofilm formation is considered one of the principal mechanisms supporting this environmental persistence because the extracellular polymeric matrix protects bacterial cells from environmental stresses and reduces the effectiveness of conventional cleaning and sanitation procedures. Although biofilm formation has frequently been associated with antimicrobial resistance, current evidence indicates that these traits are not consistently linked and are probably governed by distinct biological mechanisms. Consequently, persistence should be interpreted as the result of interactions among biofilm formation, stress-response systems, and strain-specific genetic characteristics rather than antimicrobial resistance alone [48,49,50].

The relationship between environmental persistence, biofilm formation, and antimicrobial resistance should also be interpreted cautiously because most available studies are based on cross-sectional or experimental designs that do not permit causal inferences. Furthermore, the heterogeneity of study designs and experimental models limits direct comparisons and reduces the generalizability of these findings to commercial food-processing environments.

From a food safety perspective, the persistence of biofilm-forming *L. monocytogenes* strains presents major challenges for the food industry. Contamination of raw materials, food-processing environments, or finished products may result in product recalls, production interruptions, substantial economic losses, and reduced consumer confidence [30,48]. Strengthening environmental monitoring programs, periodically validating cleaning and sanitation procedures, implementing robust Hazard Analysis and Critical Control Point (HACCP) systems, and promoting good manufacturing practices throughout the food chain remain fundamental measures for minimizing contamination risks and supporting food safety [51].

One of the principal strengths of this scoping review is the integration of evidence on the prevalence and phenotypic antimicrobial resistance of *L. monocytogenes* in animal-derived foods. By synthesizing information on resistance profiles, their distribution across food matrices, and the principal genetic determinants associated with antimicrobial resistance, this review provides a comprehensive overview that may support the early detection of emerging resistant strains, improve microbiological risk assessment, and strengthen antimicrobial resistance surveillance [3,4].

The findings also have practical implications for food safety management. Integrating information on antimicrobial resistance profiles, contamination sources, persistence mechanisms, and environmental reservoirs of *L. monocytogenes* can support the implementation and continuous improvement of internationally recognized food safety management systems, including Hazard Analysis and Critical Control Point (HACCP), ISO 22000, and FSSC 22000 [3,16,51]. Moreover, this evidence may assist in identifying critical control points, optimizing environmental monitoring programs, and strengthening the verification and validation of preventive measures throughout the food production chain.

Reducing *L. monocytogenes* contamination and limiting antimicrobial resistance require integrated interventions across the food chain, including prudent antimicrobial use in food-producing animals, continuous environmental monitoring, rigorous hygiene and sanitation programs, and robust food safety management systems [3,28,52]. Together, these measures reduce selective pressure, limit the dissemination of resistant strains, and minimize food contamination.

Although *L. monocytogenes* was not included among the priority antimicrobial-resistant pathogens in the 2024 update of the World Health Organization priority pathogens list [53], the findings of this review indicate that it remains a pathogen of considerable importance because of its foodborne transmission, persistence in food-processing environments through biofilm formation, and the occurrence of clinically relevant antimicrobial resistance profiles [2,3,27]. Strengthening microbiological and antimicrobial resistance surveillance, reinforcing food safety management systems, and promoting prudent antimicrobial use therefore remain essential strategies for reducing its impact [54].

This scoping review has several limitations inherent to the available literature. Considerable heterogeneity among the included studies with respect to food matrices, geographical regions, isolation and identification methods, antimicrobial susceptibility testing panels, and interpretative criteria limited direct comparisons across investigations. In addition, variability in the methods used to detect antimicrobial resistance determinants, together with incomplete reporting in some studies, reduced the consistency of the evidence synthesis.

Given the exploratory nature of this scoping review and the methodological heterogeneity of the available evidence, quantitative comparisons and pooled estimates of antimicrobial resistance were not feasible. Nevertheless, the findings provide a comprehensive synthesis of current knowledge, identify important evidence gaps, and highlight the need for standardized methodologies and integrated phenotypic and molecular surveillance to improve the understanding of antimicrobial resistance in *L. monocytogenes* from animal-derived foods.

## 5. Conclusions

This scoping review synthesized the available evidence on the prevalence of *L. monocytogenes* and its phenotypic antimicrobial resistance profiles in animal-derived foods and ready-to-eat products. The findings demonstrate that *L. monocytogenes* remains widely distributed across diverse food matrices, particularly meat, dairy products, and ready-to-eat foods, highlighting its continued relevance for food safety and public health.

Although most *L. monocytogenes* isolates remained susceptible to first-line antimicrobials, the detection of β-lactam- and tetracycline-resistant strains, together with the occurrence of multidrug-resistant isolates, indicates that antimicrobial resistance continues to represent an important challenge for food safety. In addition, the ability of *L. monocytogenes* to form biofilms may contribute to its persistence in food-processing environments and potentially facilitate recurrent contamination, underscoring the importance of implementing integrated prevention, monitoring, and control strategies throughout the food chain.

This scoping review provides a scientific foundation for strengthening epidemiological and antimicrobial resistance surveillance, as well as the implementation and continuous improvement of food safety management systems. The synthesized evidence can support microbiological risk assessment, inform decision-making by public health authorities and the food industry, and guide the development of evidence-based preventive strategies within a One Health framework. Collectively, the findings expand the current understanding of antimicrobial resistance in *L. monocytogenes* and provide a scientific basis for strengthening food safety policies and antimicrobial resistance surveillance.

## Figures and Tables

**Table 1:** Search Strategies for electronic databases.

Database	Search Strategy
PubMed	(“Antibiotic Resistance” OR “Antibiotic Resistance, Microbial” OR “Antimicrobial Drug Resistance” OR “Antimicrobial Drug Resistances” OR “Antimicrobial Resistance, Drug” OR “Antimicrobial Resistances, Drug” OR “Drug Antimicrobial Resistance” OR “Drug Antimicrobial Resistances” OR “Drug Resistance, Microbial” OR “Drug Resistances, Microbial” OR “Resistance, Antibiotic” OR “Resistance, Drug Antimicrobial” OR “Resistances, Drug Antimicrobial”) AND (“*Listeria monocytogenes*”) AND (“Disease, Food-borne” OR “Disease, Foodborne” OR “Food borne Disease” OR “Food borne Diseases” OR “Food borne Illness” OR “Food borne Illnesses” OR “Food Poisoning” OR “Food Poisonings” OR “Food-borne Disease” OR “Food-borne Diseases” OR “Food-borne Illness” OR “Food-borne Illnesses” OR “Foodborne Disease” OR “Foodborne Diseases” OR “Foodborne Illness” OR “Foodborne Illnesses” OR “Illness, Food-borne” OR “Illness, Foodborne” OR “Illnesses, Foodborne” OR “Poisoning, Food”)
Scopus	TITLE-ABS-KEY((“Antibiotic Resistance” OR “Antibiotic Resistance, Microbial” OR “Antimicrobial Drug Resistance” OR “Antimicrobial Drug Resistances” OR “Antimicrobial Resistance, Drug” OR “Antimicrobial Resistances, Drug” OR “Drug Antimicrobial Resistance” OR “Drug Antimicrobial Resistances” OR “Drug Resistance, Microbial” OR “Drug Resistances, Microbial” OR “Resistance, Antibiotic” OR “Resistance, Drug Antimicrobial” OR “Resistances, Drug Antimicrobial”)) AND TITLE-ABS-KEY((“*Listeria monocytogenes*”)) AND TITLE-ABS-KEY((“Disease, Food-borne” OR “Disease, Foodborne” OR “Food borne Disease” OR “Food borne Diseases” OR “Food borne Illness” OR “Food borne Illnesses” OR “Food Poisoning” OR “Food Poisonings” OR “Food-borne Disease” OR “Food-borne Diseases” OR “Food-borne Illness” OR “Food-borne Illnesses” OR “Foodborne Disease” OR “Foodborne Diseases” OR “Foodborne Illness” OR “Foodborne Illnesses” OR “Illness, Food-borne” OR “Illness, Foodborne” OR “Illnesses, Foodborne” OR “Poisoning, Food”))
Web of Science	(“Antibiotic Resistance” OR “Antibiotic Resistance, Microbial” OR “Antimicrobial Drug Resistance” OR “Antimicrobial Drug Resistances” OR “Antimicrobial Resistance, Drug” OR “Antimicrobial Resistances, Drug” OR “Drug Antimicrobial Resistance” OR “Drug Antimicrobial Resistances” OR “Drug Resistance, Microbial” OR “Drug Resistances, Microbial” OR “Resistance, Antibiotic” OR “Resistance, Drug Antimicrobial” OR “Resistances, Drug Antimicrobial”)) AND (“*Listeria monocytogenes*”) AND (“Disease, Food-borne” OR “Disease, Foodborne” OR “Food borne Disease” OR “Food borne Diseases” OR “Food borne Illness” OR “Food borne Illnesses” OR “Food Poisoning” OR “Food Poisonings” OR “Food-borne Disease” OR “Food-borne Diseases” OR “Food-borne Illness” OR “Food-borne Illnesses” OR “Foodborne Disease” OR “Foodborne Diseases” OR “Foodborne Illness” OR “Foodborne Illnesses” OR “Illness, Food-borne” OR “Illness, Foodborne” OR “Illnesses, Foodborne” OR “Poisoning, Food”)
Embase	(‘antibacterial drug resistance’ OR ‘antibacterial resistance’ OR ‘antibiotic non-susceptibility’ OR ‘antibiotic nonsusceptibility’ OR ‘antimicrobial drug resistance’ OR ‘antimicrobial resistance’ OR ‘bacterial drug resistance’ OR ‘bacterial resistance’ OR ‘bacterium resistance’ OR ‘drug resistance, bacterial’ OR ‘drug resistance, microbial’ OR ‘microbial drug resistance’ OR ‘resistance, antibiotic’ OR ‘antibiotic resistance’) AND (‘*bacterium monocytogenes*’ OR ‘*Corynebacterium infantisepticum*’ OR ‘*Corynebacterium parvulum*’ OR ‘*Erysipelothrix monocytogenes*’ OR ‘*Listerella hepatolytica*’ OR ‘listeriosis monocytogenes’ OR ‘*Listeria monocytogenes*’) AND (‘food borne disease’ OR ‘food borne diseases’ OR ‘food borne illness’ OR ‘food borne illnesss’ OR ‘food borne infection’ OR ‘food borne infections’ OR ‘food infection’ OR ‘food intoxication’ OR ‘food toxicity’ OR ‘food toxicology’ OR ‘foodborne disease’ OR ‘foodborne diseases’ OR ‘foodborne illness’ OR ‘foodborne illnesses’ OR ‘foodborne infection’ OR ‘foodborne infections’ OR ‘food poisoning’)

**Table 2:** Inclusion and Exclusion Criteria for Study Selection.

Inclusion Criteria	Exclusion Criteria
Original observational studies reporting primary data.	Studies focusing on microorganisms other than *L. monocytogenes*.
Studies reporting the isolation and identification of *L. monocytogenes* from food matrices.	Experimental laboratory studies using reference strains or artificially inoculated samples.
Studies evaluating the antimicrobial susceptibility or resistance profiles of *L. monocytogenes* isolates.	Narrative reviews, systematic reviews, meta-analyses, editorials, letters to the editor, conference abstracts, case reports, book chapters, and other publications not reporting original primary research data.
Studies conducted on meat, meat products, raw milk, dairy products, and ready-to-eat animal-derived foods.	Studies that did not report antimicrobial susceptibility or resistance testing results.
Studies providing sufficient methodological detail, microbiological identification, and antimicrobial susceptibility testing.	Studies focusing exclusively on environmental samples unrelated to food matrices.

**Table 3:** Characteristics of the included studies.

Reference	Year	Country	Sample Size (*n*)	Food Matrix
[13]	2014	Nigeria	300	Raw beef, goat and chicken meat, and meat products
[14]	2015	Rumania	144	Raw poultry meat
[15]	2017	Iran	442	Raw meat from different animal sources, meat products, milk, and dairy products
[16]	2022	India	15	Ready-to-eat foods
[18]	2025	Portugal	75	Raw beef, raw pork, meat products, and meat preparations
[21]	2017	India	457	Raw bovine milk
[17]	2017	India	765	Raw beef
[22]	2018	Iran	545	Raw milk and dairy products
[24]	2021	Ethiopia	450	Raw beef
[25]	2025	Ethiopia	100	Raw beef
[23]	2019	Egypt	100	Raw milk

**Table 4:** Prevalence of *L. monocytogenes* in different food matrices.

Reference	Year	Food Matrix	Sample Size (*n*)	Positive Samples	Reported Prevalence (%)
[13]	2014	Beef, goat, chicken meat, suya, Kilishi, Balangu, and Tsire	300	12	4
[14]	2015	Raw chicken meat	144	19	13.2
[15]	2017	Sausages, pasteurized milk, cheese, raw chicken meat, beef, goat, and sheep meat cubes	267	8	3
[16]	2022	Chicken burger, vegetarian paneer burger, egg roll, vegetable-filled Momos, vegetable spring roll, cheeseburger, chicken Chow mein, cheese salad, white pasta, fried potato chaat, chicken curry, chicken nuggets, vegetable cutlets, chicken spring roll, and Aloo paratha.	15	9	60
[18]	2025	Raw beef, raw pork, meatballs, hamburgers, fresh sausages, breaded meat skewers, Alheira sausage, and Moura sausage	75	12	16
[21]	2017	Raw bovine milk	457	5	1.1
[17]	2017	Raw beef	765	1	0.1
[22]	2018	Raw milk, cheese, butter, curd, and ice cream	545	22	4
[24]	2021	Raw beef	450	20	4.4
[25]	2025	Raw beef	100	2	2
[23]	2019	Raw bovine milk	100	7	7

For studies that evaluated multiple food matrices, only the food matrices meeting the eligibility criteria of the present review were included in this table.

**Table 5:** Reported antimicrobial resistance profiles of *L. monocytogenes*.

Reference	Year	Food Matrix	*L. monocytogenes* Isolates Evaluated	Main Resistance Findings	Main Susceptibility Findings	MDR Profile
[13]	2014	Beef, goat, chicken meat, suya, Kilishi, Balangu, and Tsire	12	100% resistant to 9/14 antimicrobial agents; recurrent resistance to penicillin, SXT, nitrofurantoin, tetracycline, streptomycin, ampicillin, lincomycin, and trimethoprim	Susceptible to gentamicin; most isolates were susceptible to kanamycin and ciprofloxacin	Yes
[14]	2015	Raw chicken meat	19	Ampicillin (10.52%); sulfamethoxazole (5.26%); sulfamethoxazole/trimethoprim (5.26%); nalidixic acid (10.52%); ciprofloxacin (10.52%); tetracycline (31.57%); chloramphenicol (21.05%).	100% susceptible to the aminoglycosides tested	23% MDR
[15]	2017	Sausages, pasteurized milk, cheese, raw chicken meat, and beef, goat, and sheep meat cubes	22	Resistance to penicillin G (27.2%)	Susceptible to chloramphenicol, linezolid, amoxicillin/clavulanic acid, kanamycin, tetracycline, SXT, gentamicin, and ampicillin	No
[16]	2022	Chicken burger, veg paneer burger, egg roll, vegetable filling for Momos, veg spring roll, cheese burger, chicken Chowmein, cheese salad, white pasta, fried potato chaat, chicken curry, chicken nuggets, veg cutlets, chicken spring roll and Aloo paratha.	Not clearly specified	Levofloxacin (80%); cefotaxime (80%); ciprofloxacin (80%); cotrimoxazole (60%); gentamicin, azithromycin, and chloramphenicol (40%); oxacillin (20%)	Not clearly specified	Yes
[18]	2025	Raw beef, raw pork, meatballs, hamburgers, fresh sausages, breaded meat skewers, Alheira sausage, and Moura sausage	21	SXT (76.19%); ciprofloxacin (38.10%); meropenem (33.33%); tetracycline and erythromycin (28.57%); rifampicin (23.8%); kanamycin (14.3%)	100% susceptible to ampicillin, chloramphenicol, gentamicin, linezolid, and vancomycin	28.6% MDR
[21]	2017	Raw bovine milk	5	100% resistant to penicillin G, piperacillin, oxacillin, and ceftriaxone; 80% to ampicillin, amoxicillin/clavulanic acid, and nalidixic acid; 60% to ceftazidime	100% susceptible to vancomycin, ciprofloxacin, gatifloxacin, amikacin, azithromycin, gentamicin, kanamycin, norfloxacin, and streptomycin	Yes
[17]	2017	Raw beef	1	Resistant to clindamycin and gentamicin (100%).	Susceptible to amoxicillin, ampicillin, ceftazidime, cefuroxime, chloramphenicol, and doxycycline	Not
[22]	2018	Raw milk, cheese, butter, curd, and ice cream	22	Clindamycin (22.7%), erythromycin (36.3%), kanamycin (9.0%), streptomycin (45.4%), tetracycline (86.3%), chloramphenicol (77.2%), gentamicin (18.1%), penicillin (77.2%), rifampin (4.5%), and amoxicillin/clavulanic acid (13.6%).	100% susceptible to vancomycin and SXT	Frequent (≥2–3 antimicrobial agents)
[24]	2021	Raw beef	20	Oxacillin (80%); cefotaxime (75%); amikacin and nalidixic acid (70%); chloramphenicol (60%); tetracycline (55%)	Amoxicillin (95%); vancomycin (90%); clindamycin (85%)	95% MDR
[25]	2025	Raw beef	2	Cloxacillin and nalidixic acid (100%); penicillin (76%); tetracycline (59%); chloramphenicol (24%)	Susceptible to amoxicillin, vancomycin, sulfamethoxazole, gentamicin, ampicillin, chloramphenicol, and tetracycline	No
[23]	2019	Raw bovine milk	17	Oxytetracycline (100%); penicillin G (94.12%); neomycin (94.12%); nalidixic acid (88.24%); kanamycin (82.35%); chloramphenicol (76.47%); erythromycin (70.59%); amoxicillin (70.59%); streptomycin (58.82%); sulfamethoxazole/trimethoprim (52.94%); ciprofloxacin (41.18%); ampicillin (23.53%); gentamicin (5.88%); norfloxacin (5.88%)	Highest susceptibility to norfloxacin	82% resistant to ≥5 antimicrobial agents

The number of isolates evaluated may differ from the number of positive samples, as some studies recovered more than one isolate from a single positive sample. Abbreviations follow the terminology used in the original studies (SXT: trimethoprim/sulfamethoxazole; MDR: multidrug resistance). MDR data are presented as reported by the original studies. Definitions of MDR, antimicrobial panels, susceptibility testing methods, and interpretative criteria varied across studies; therefore, MDR frequencies should not be directly compared.

## Data Availability

The data analyzed in this study are fully synthesized and available within the body of the manuscript and its accompanying tables.

## Supplementary Materials

The following supporting information can be downloaded at https://www.mdpi.com/article/10.3390/foods15183274/s1, File S1: Optimized_Matrix_Listeria; File S2: Scoping Review Resistance *L. monocytogenes*, File S3: PRISMA Sr 2020 checklist.

## Abbreviations

AMR	Antimicrobial Resistance
DeCS	Health Sciences Descriptors
MDR	Multidrug Resistance
MeSH	Medical Subject Headings
OSF	Open Science Framework
PCC	Population–Concept–Context
PRISMA	Preferred Reporting Items for Systematic Reviews and Meta-Analyses
PRISMA-ScR	Preferred Reporting Items for Systematic Reviews and Meta-Analyses Extension for Scoping Reviews
RTE	Ready-to-Eat
SXT	Trimethoprim/Sulfamethoxazole
HACCP	Hazard Analysis and Critical Control Point
JBI	Joanna Briggs Institute
CLSI	Clinical and Laboratory Standards Institute
EUCAST	European Committee on Antimicrobial Susceptibility Testing
